# Expression of ferroptosis-related gene correlates with immune microenvironment and predicts prognosis in gastric cancer

**DOI:** 10.1038/s41598-022-12800-6

**Published:** 2022-05-24

**Authors:** Siyuan Song, Peng Shu

**Affiliations:** 1 Affiliated Hospital of Nanjing University of Chinese Medicine, Nanjing, 210029 Jiangsu Province China; 2 Nanjing University of Chinese Medicine, Nanjing, 210029 Jiangsu Province China; 3 Jiangsu Provincial Hospital of Chinese Medicine, Nanjing, 210029 Jiangsu Province China

**Keywords:** Cancer, Cancer microenvironment, Gastrointestinal cancer

## Abstract

The study is to explore the role of ferroptosis-related genes (FRGs) in the occurrence and development of gastric cancer (GC), and to construct a new prognosis signature to predict the prognosis in GC. Clinical information and corresponding RNA data of GC patients were downloaded from TCGA and GEO databases. Consensus clustering was performed to identify new molecular subgroups. ESTIMATE, CIBERSORT, McpCounter and TIMER algorithm were used to analyze the infiltration of immune cells in two molecular subgroups. LASSO algorithm and multivariate Cox analysis were used to construct a prognostic risk signature. Functional analysis was conducted to elucidate the underlying mechanisms. Finally, the FRPGs were verified by Quantitative Real-Time PCR. We obtained 16 FRGs and divided GC patients into two subgroups by consistent clustering. Cluster C1 with a higher abundance of immune cell infiltration but lower probability in response to immunotherapy, it was reasonable to speculate that Cluster C1 was in accordance with the immune rejection type. Functional analysis showed that the biological process of DEGs in training cohort mainly included immune globulin, and human immune response mediated by circulating immune globulin. GSEA analysis showed that compared with Cluster C2, Cluster C1 showed lower expression in lipid metabolism. The nomogram combined with risk signature and clinical features can accurately predict the prognosis of GC patients. We identified two molecular subtypes, Clusters C1 and C2. In Cluster C1, patients with poor prognosis present with a hyperimmune status and low lipid metabolism, and we speculate that Cluster C1 was in accordance with the immune rejection type. The risk model based on FRPGs can accurately predict the prognosis of GC. These results indicated that ferroptosis is associated with TIME, and deserved considerable attention in determining immunotherapy treatment strategy for GC patients.

## Introduction

A preprint has previously been published^1^.

Gastric cancer (GC) is the fourth leading cause of cancer-related death^2^. The overall survival of patients with GC varies widely in different regions of the world. For example, the 5-year survival rate is 31% in the United States, 19% in the United Kingdom, and 26% in Europe^3^. Because the early stage of GC is usually asymptomatic, it is mostly late when it is discovered, resulting in a 5-year overall survival rate (OS) of less than 40%^4^. Therefore, exploring new and more effective treatment methods has become a problem to be solved.

The occurrence and development of tumors are closely related to cell death. Ferroptosis is a form of non-apoptotic cell death found in recent years^5^. Triggered by lipid reactive oxygen species (ROS)^6^. Ferroptosis is related to the occurrence of many kinds of tumors, including lung cancer^7^, breast cancer^8^, colorectal cancer^9^, and GC^10^. In recent years, prognosis models of ferroptosis associated with various diseases have been constructed, such as adrenocortical carcinoma^11^, and pancreatic cancer^12^. Many ferroptosis genes including GPX4, SLC7A11 and NRF2 have been found to be promising targets for inducing tumor cell death. For example, an innovative NRF2 nano-modulator induces lung cancer ferroptosis and elicits an immunostimulatory tumor microenvironment^13^. SLC7A11 promoted the proliferation, migration, and invasion of renal cancer cells by enhancing GPX4 output, which in turn inhibits ferroptosis^14^. Therefore, targeting ferroptosis may be a new strategy for cancer treatment.

Tumor immunotherapy, as a new treatment method based on human immune system, plays an anti-tumor role by immune regulation. The use of checkpoint inhibitors has been proved to be of great significance in improving the objective remission rate of tumors and prolonging the survival time of patients^15,16^. In the last decade, molecular subtype-based classification of GC offers opportunities for personalized treatment. Biomarkers, particularly microsatellite instability (MSI), programmed cell death ligand 1 (PD-L1), human epidermal growth factor receptor 2 (HER2), tumor mutational burden, and Epstein-Barr virus. The use of for first-line immunotherapy for advanced GC, HER2- patients can choose Nivolumab or Sintilimab combined with chemotherapy, and HER2 + patients are recommended to be treated with Trastuzumab combined with Pembrolizumab combined with chemotherapy. In addition to immune checkpoint inhibitors, cellular immunotherapy may become another effective weapon for the treatment of advanced GC^17^. Tumor infiltrating immune cells (TIIC) are related to many kinds of tumor prognosis and immunotherapy response^18–20^. For example, M2 macrophages are enriched in bladder cancer tissue, which can be used as a potential immunotherapy target for bladder cancer^21^. Many kinds of tumor immunotherapy related to natural killer cells (NK) have also entered the clinical trial stage^22^. It has been proved that TIME is closely related to the pathogenesis of GC^23^. Therefore, mining immune-related ferroptosis targets is an effective way to optimize tumor immunotherapy^24^.

**Figure 1:**
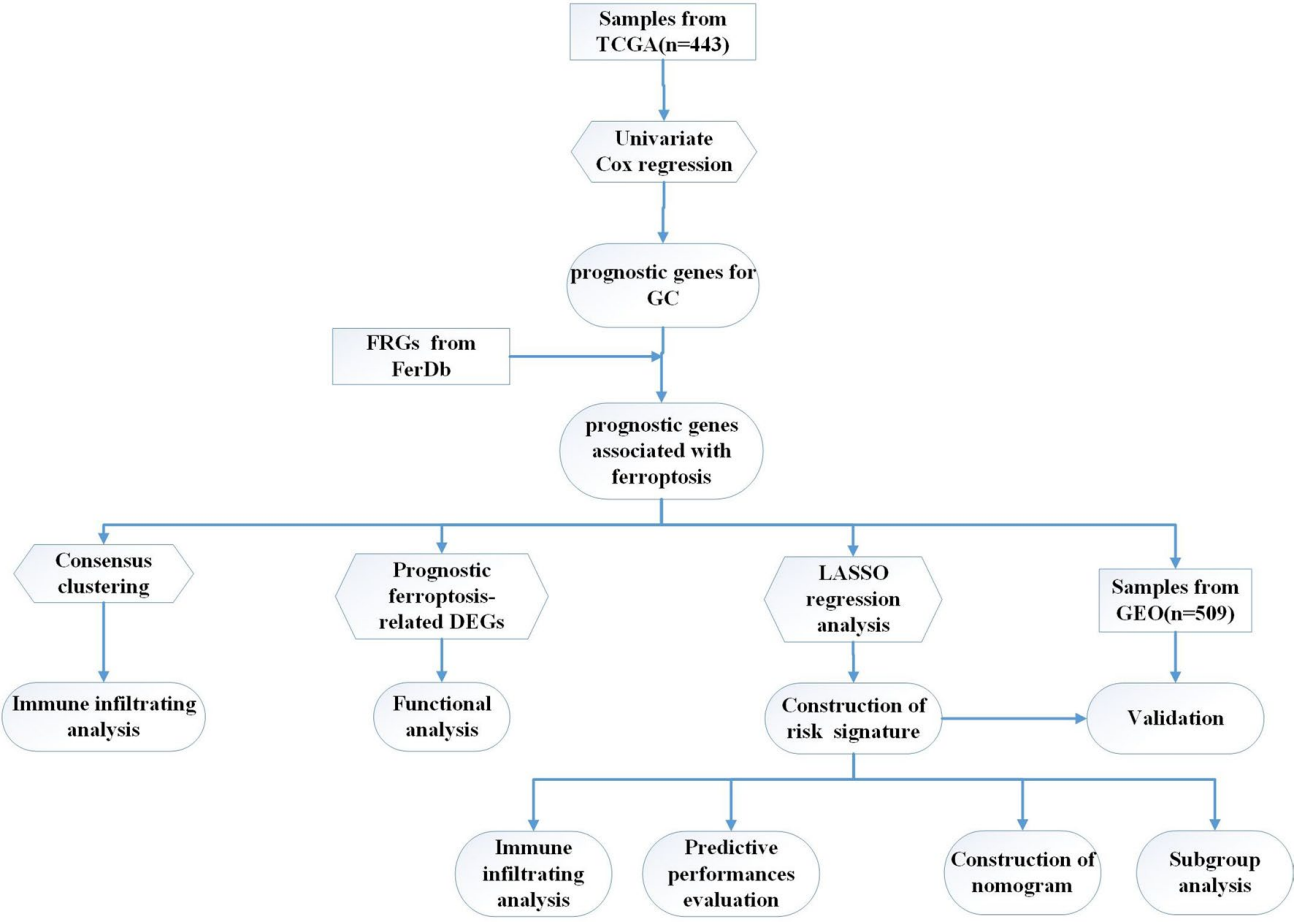
The protocol of our study procedures.

In this study, we comprehensively analyzed the FRGs to explore the influence of ferroptosis on the TIME and survival of patients with GC. In addition, we constructed a FRGs-risk signature to evaluate the prognostic value in GC, which will provide a new strategy for targeted and individualized treatment of GC. The protocol of our study procedures is shown in Fig. 1.

## Method

### Data collection and analysis

The FPKAPLAN-MEIER ANALYSIS gene expression profile of GC (TCGA-STAD) was obtained from TCGA database (https://portal.gdc.cancer.gov/). As a training cohort, we obtained 443 clinical samples, including 375 samples of cancer tissues and 68 samples of adjacent tissues. We extracted the mapping information of GeneSymbol and ENSG_ID, mapped the ENSG_ID to GeneSymbol, and when there were multiple matches, took the median, and finally obtained the transformed expression spectrum. Finally, the data was further standardized by log2(X + 1) transformation and we used samples with complete clinical information for subsequent analysis. The microarray data GSE84426 and GSE84437 were downloaded from GEO database (http://www.ncbi.nlm.http://nih.gov/geo/) through GPL6947 application platform. As validation cohorts, we obtained 509 GC tissue samples. Clinical information of the above patient is shown in Table 1. 259 genes related to ferroptosis were collected from FerrDB (http://www.zhounan.org/ferrdb/) database^25^, including driver, suppressor, and marker, Species was defined as Human.

**Table 1:** Clinical characteristic of the GC patient used in this study.

	TCGA	GEO
No. of patients	443	509
**Age (%)**		
≤ 65	246 (55.5)	325 (63.8)
> 65	197 (44.5)	184 (36.2)
**Gender (%)**		
Female	158 (35.6)	159 (31.2)
Male	285 (64.4)	350 (68.8)
**Grade (%)**		
G1	12 (2.7)	NA
G2	159 (35.9)	NA
G3	263 (59.4)	NA
GX	9 (2)	NA
**Stage (%)**		
I	59 (13.3)	NA
II	97 (21.9)	NA
III	183 (41.3)	NA
IV	44 (23.5)	NA
**Survival status**		
OS day(median)	413	172
**Ending (%)**		
Survival	270 (60.9)	265 (52.1)
Death	173 (39.1)	244 (47.9)

NA *not available.*

### Identification of molecular subtype

The genes related to the prognosis of GC were screened out by univariate Cox analysis. We intersected the prognosis-related genes of GC with ferroptosis genes by Venn diagram to obtain ferroptosis-related prognosis genes (FRPGs). Cluster analysis was performed using ConsensusClusterPlus, using agglomerative pam clustering with a 1-pearson correlation distances and resampling 80% of the samples for 10 repetitions. The optimal number of clusters was determined using the empirical cumulative distribution function plot.

### Immune analysis

Immunological analysis was used to explore the immune differences between the two subgroups. Estimate algorithm (estimation of violent and immune cells in malignant tumor organization using expression) is used to evaluate the proportion of immune-matrix components in TIME, include Stromal Score (reflecting the presence of matrix), Immune Score (reflecting the level of immune cell infiltration) and ESTIMAT Score (comprehensive score of immunity and matrix)^26^. The higher the corresponding score, the larger the proportion of corresponding components in TIME. Using MCP Counter and TIMER databases^27^ to calculate the abundance of immune infiltrating cells. CIBERSORT algorithm was used to estimate the data of tumor infiltrating immune cells ^28^.

### Establishment of prognostic risk signature based on FRPGs

The “glmnet” R package^29^ was used for LASSO analysis to further select hub prognostic markers, and the minimum lambda was defined as the optimal value. According to the risk score of the prognosis signature, GC patients were divided into high and low risk groups. Kaplan–Meier survival curve and time-dependent ROC curve were used to analyze and compare the survival situation between the two groups. Immunohistochemical (IHC) staining of hub FRPGs were examined by human protein atlas (HPA) (https://www.proteinatlas.org/about/download). Maftools was used to calculate the mutation of hub FRPGs, and “ggplot2” package^30^ was used to draw the mutation distribution map.

### Validation of prognostic risk signature

Multivariate Cox analysis and subgroup analysis were used to evaluate the independence of the prognosis signature and the clinical characteristics (including Gender, Age,Grade, and Stage) of patients. The GEO validation cohort was used to verify the accuracy of the established prognosis signature. Combining the prognosis signature and clinical features, nomogram was constructed to predict the 1,3 and 5-year survival rate of GC patients.

### Functional enrichment analysis

Differentially expressed genes (DEGs) between the two clusters were identified using R package ‘‘Limma’’^31^ in the training cohort, the false discovery rate (FDR) was less than 0.05, and the difference multiple was 1.5 times as the screening standard. GSEA enrichment analysis was carried out according to DEGs. Metascape software was used to construct PPI network of DEGs^32^. Moreover, we also performed survival analysis on samples in the GEO validation cohorts, divided the samples into high and low risk groups according to risk scores, and screened out DEGs by “limma” R package^31^. Gene Ontology (GO) analysis and Kyoto Encyclopedia of Genes and Genomes (KEGG) analysis^33,34^ were performed to enrich associated pathways.

### Cell culture

GC cell lines HGC-27 and normal human gastric epithelial cell lines GSE-1 were purchased from Nanjing KGI Biotechnology Company. All cells were cultured in RPMI-1640 medium supplemented with 5% fetal bovine serum at 37 °C in a humidified atmosphere with 5% CO2.

### Quantitative real-time PCR

Total RNA was extracted from cells using the TRIzol kit according to the manufacturer's protocol. Reverse transcription was performed using the PrimeScript RT kit (Takara, China) according to the manufacturer's instructions. SYBR PrimeScript RT-PCR Kit (Takara) is used for quantitative reverse transcription polymerase chain reaction (qRT-PCR) analysis. The 2^−ΔΔCt^ statistic was used to calculate the expression level of the gene. The specific sequences of the different primers used in this study are included in Supplementary Table S1.

### Statistical analysis

All statistical analysis in this paper was carried out by R software and GraphPad Prism. Student’s t-test was used for statistical analysis between two groups, and one-way ANOVA analysis was selected flexibly when there were three or more groups. *P* < 0.05 was considered to be statistically significant.

### Statement

All methods were carried out in accordance with relevant guidelines and regulations. Ethical approval is not applicable for this study.

## Results

### Identification of molecular subtypes based on FRGs

**Figure 2:**
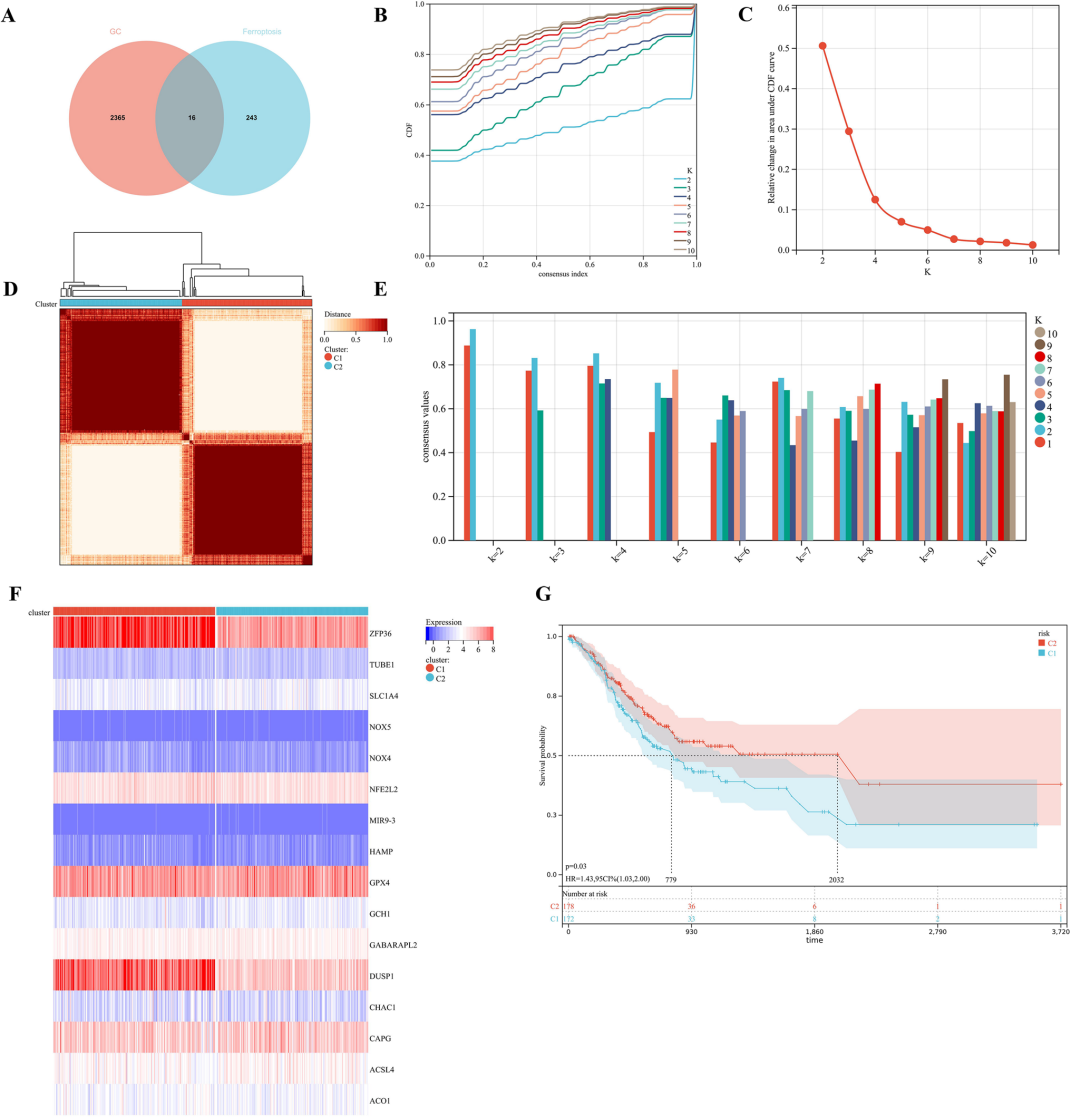
Molecular subtype identification based on FRGs. (**A**) Venn diagram, a prognostic gene for GC associated with ferroptosis. The pink represents GC-related genes, blue represents ferroptosis-related genes. (**B**–**E**) K = 2 was identified the optimal value for consensus clustering. (**B**) Represents Cumulative distribution curve. (**C**) Represents Area under distribution curve. (**D**) represents Heatmap of consensus clustering. (**E**) Represents Sample clustering consistency. (**F**) Heatmap of FRPGs in two subgroups. (**G**) Kaplan–Meier survival curves for two subgroups.

Through univariate Cox analysis, we obtained 2,381 GC prognosis-related genes, and 16 FRPGs were obtained by intersecting 2,381 GC prognosis-related genes with 259 ferroptosis genes (Fig. 2A). The consensus clustering approach was conducted to divide the GC patients in the training cohort. The optimal clustering stability was identified when K = 2 (Fig. 2B–E). Cluster C1 included 210 patients, while Cluster C2 included 197 patients. The two subgroups were visualized by heatmap (Fig. 2F). Kaplan–Meier survival curves of two different subgroups showed that Cluster C2 showed significant median survival advantage, while Cluster C1 showed poor prognosis (Fig. 2G).

### Different TIME and immune status in the two molecular subtypes

**Figure 3:**
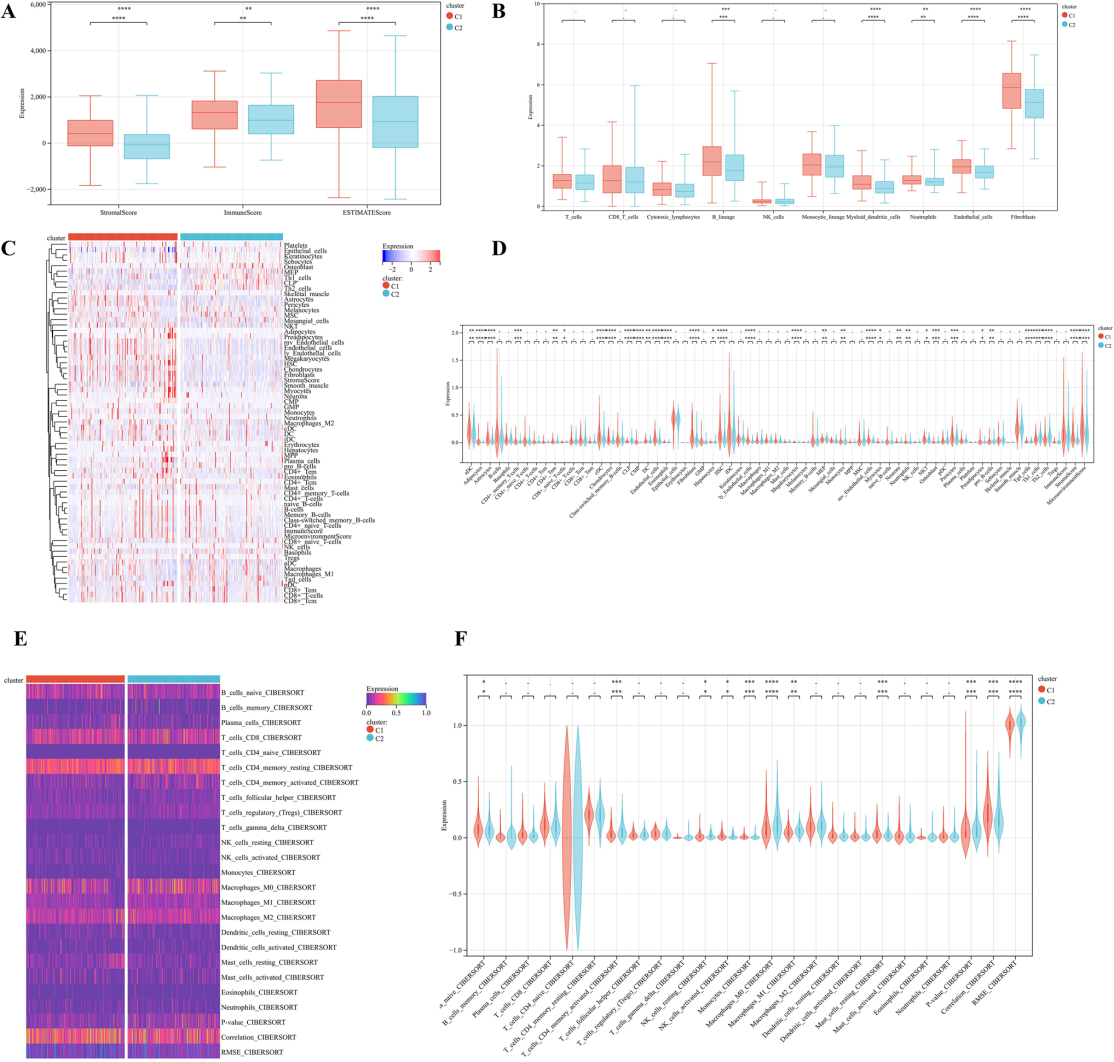
Immune analysis of two molecular subtypes. (**A**) Stromal score, Immune score, ESTIMATE score and calculated by ESTIMATE algorithm (*p < 0.05; **p < 0.01; ***p < 0.001). (**B**) Abundance of ten immune filtrating cells evaluated by MCPcounter algorithm (*p < 0.05; **p < 0.01; ***p < 0.001). (**C**) Heatmap depicting the enriching level of immune related cells evaluated by TIMER. (**D**) Statistical analysis of immune related cells evaluated by TIMER (*p < 0.05; **p < 0.01; ***p < 0.001). (**E**) Heatmap depicting the enriching level of 25 immune related cells evaluated by CIBERSORT algorithm. (**F**) Statistical analysis of 25 immune related cells evaluated by CIBERSORT algorithm (*p < 0.05; **p < 0.01; ***p < 0.001).

The ESTIMATE algorithm showed significantly higher ESTIMATE scores (*P* < 0.0001), higher Immune score (*P* < 0.01), and higher Stromal score (*P* < 0.0001) in Cluster C1 compared with Cluster C2 (Fig. 3A). The MCPCounter algorithm showed that the expression levels of B-lineage (*P* < 0.00), Myoid-Dendritic-cells (*P* < 0.0001), Neutrophils (*P* < 0.01), Endothelial-cells (*P* < 0.0001) and Fibroblasts (*P* < 0.0001) in Cluster C1 were significantly higher than those in Cluster C2 (Fig. 3B). The TIMER algorithm showed that fibrolasts (*p* = 7.80e−12), CD4 + T cells (*p* = 0.0009), and B cells (*p* = 0.0023) were significantly higher in Cluster C1 than in Cluster C2. Macrophases-M2 and Tregs in two Cluster was no significant difference (Fig. 3C,D). The CIBERSORT algorithm indicated Macrophases-M0 (*p* = 0.0000052), CD4 Memory Activated-T cells (*p* = 0.00024), Macrophases-M1 (*p* = 0.001), Nave-B-cells (*p* = 0.001), NK cells (*P* = 0.02) were significantly higher in Cluster C1 than in Cluster C2. Macrophases-M2 in two Cluster was no significant difference (Fig. 3E,F), suggesting a relatively low immune status in Cluster C2. These results demonstrated that the TIME and immune status of the two molecular subtypes differed significantly. Cluster C1 with a poor prognosis had a high immune status, so we speculate that Cluster C1 is immune rejection type.

### Establishment of prognostic risk signature based on FRPGs

**Figure 4:**
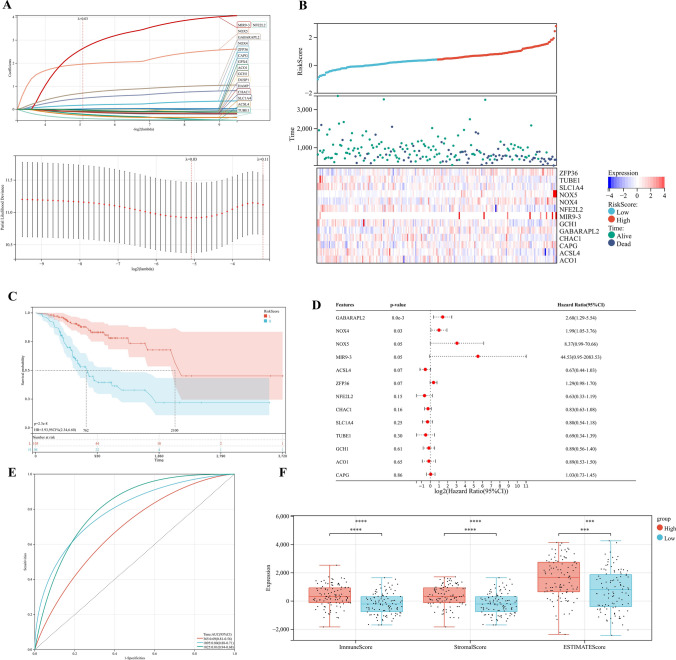

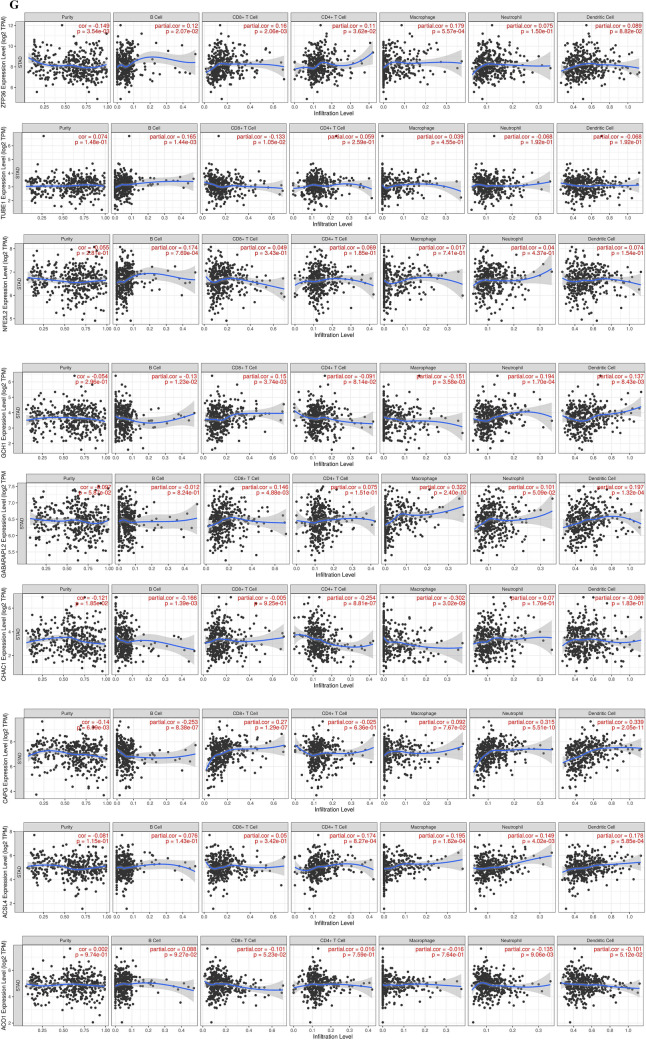

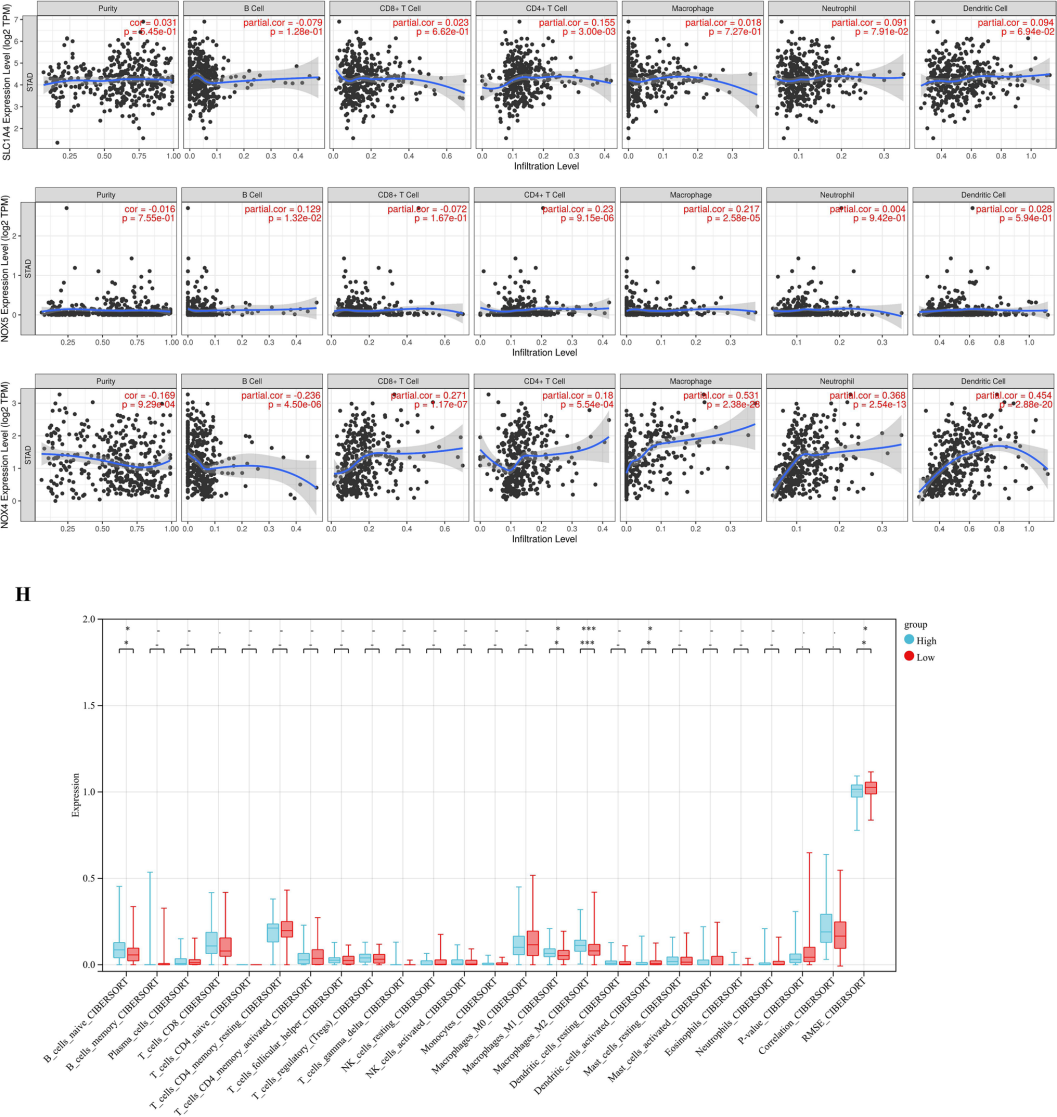
Establishment of prognostic risk signature based on FRPGs in the training cohort. (**A**) LASSO analysis with suitable lambda. (**B**) Distribution of survival status, risk score, and heatmap of GC patients in the high and low risk groups. (**C**) Survival curve of the GC patients in the two groups. (**D**) Forest map of multi-factor survival analysis. (**E**) Time-dependent ROC curve of the risk signature. (**F**) Stromal score, Immune score, and ESTIMATE score in the high and low risk groups (*p < 0.05; **p < 0.01; ***p < 0.001). (**G**) Correlation between the FRPGs and the infiltration level of immune cells in TIMER database. (**H**) Statistical analysis of immune related cells evaluated by CIBERSORT algorithm in the two groups (*p < 0.05; **p < 0.01; ***p < 0.001).

**Figure 5:**
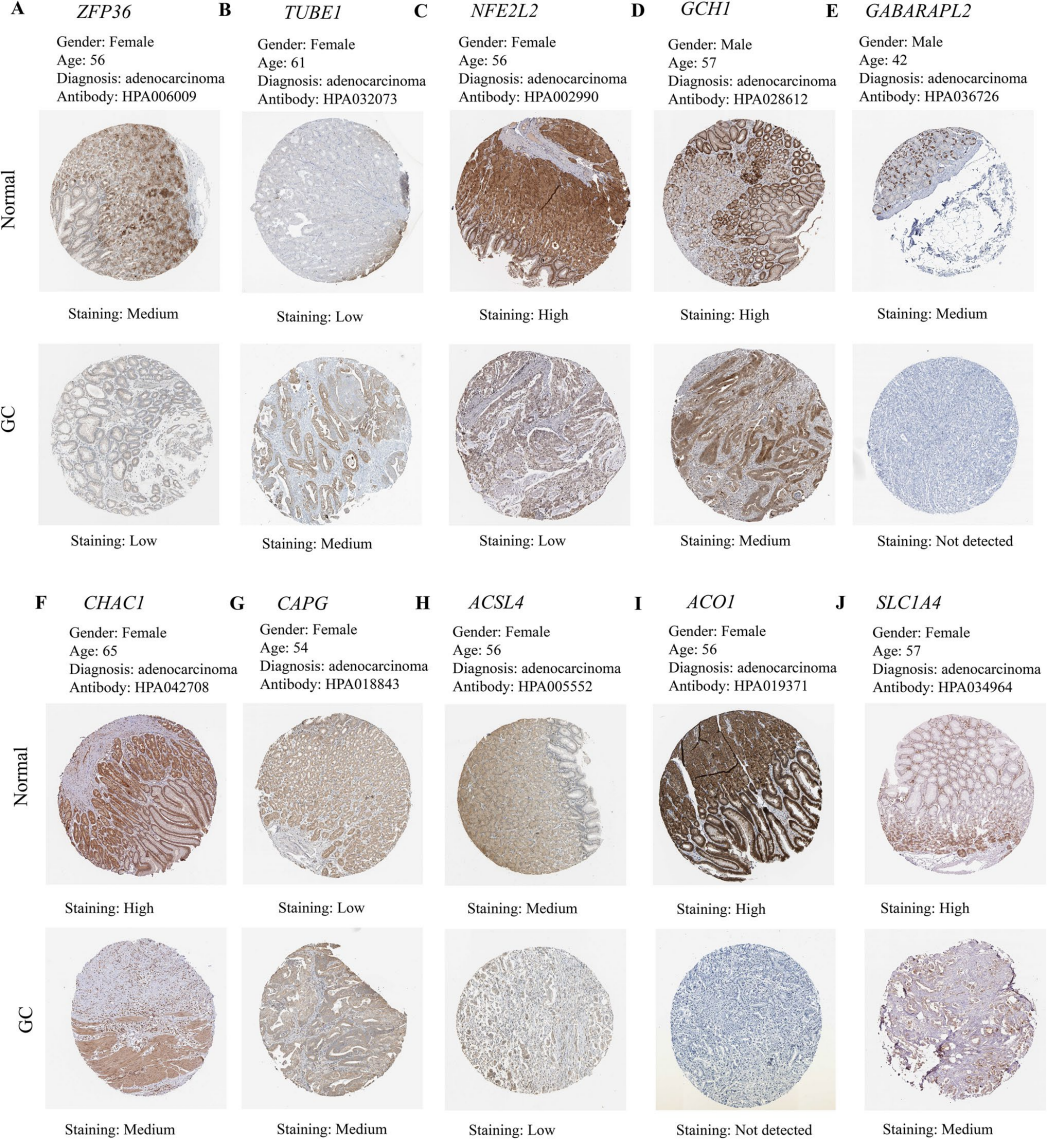
Immunohistochemical staining of FRPGs in HPA. The expression of (**A**) ZFP36, (**B**) TUBE1, (**C**) NFE2L2, (**D**) GCH1, (**E**) GABARAPL2, (**F**) CHAC1, (**G**) CAPG, (**H**) ACSL4, (**I**) ACO1, and (**J**) SLC1A4 in the HPA.

**Figure 6:**
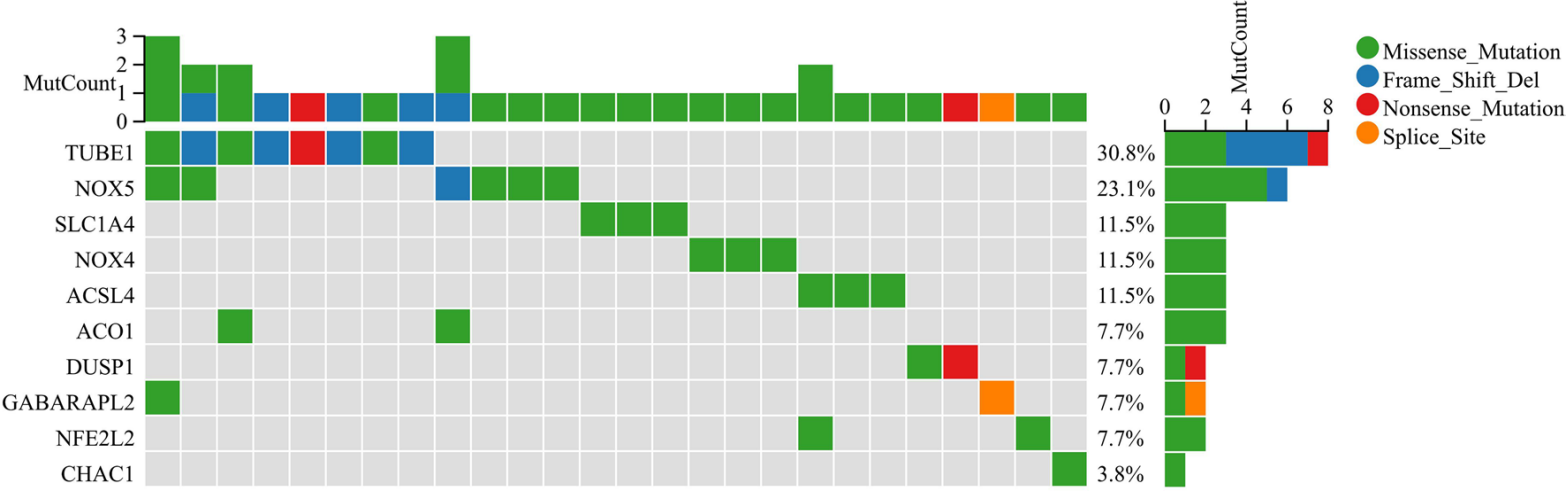
Mutations of FRPGs in the training cohort. The green represents Missense Mutation, blue represents Frame Shift Del, red represents Nonsense Mutation, and orange represents Splice Site.

We signatureed the risk based on LASSO analysis, and we set the lambda value to be 0.029411686746793, Built signature RiskScore = [0.17987688835102 * ZFP36] − [0.2111220517453 * TUBE1] − [0.1164248611519 * SLC1A4] + [1.958393356095 * NOX5] + [0.480740705518259*NOX4] − [0.39552084635819 * NFE2L2] + [2.63582963497227 * MIR9] − [0.0833648850166638*GCH1] + [0.702888544996765 * GABARAPL2] − [0.117744902736126 * CHAC1] + [0.010366289402305 * CAPG] − [0.25896183370357 * ACSL4] − [0.033599913666074 * ACO1], thirteen genes were finally obtained, and three of them were risk genes with the risk ratio greater than 1 (Fig. 4A). The established risk signature successfully classified the GC patients into high risk and low risk groups (Fig. 4B). Kaplan–Meier analysis showed that patients in the low risk group had a better overall survival than those in the high risk group (Fig. 4C). Based on the information such as the survival status and survival time of the patients, we drew the forest map for multivariate survival analysis of FRPGs (Fig. 4D). ROC analysis showed that the risk signature constructed exhibited accurate prediction ability within 5 years, with AUC of 0.69, 0.80 and 0.81 for 1, 3 and 5 years (Fig. 4E). Finally, the TIME of the two groups was evaluated using the ESTIMATE algorithm, and the results showed that the high risk group had a higher ESTIMATE score (*P* = 1.2e−4), higher Immune score (*P* = 4.8e−7), and higher Stromal score (*P* = 5.1e−7) (Fig. 4F). The TIMER database was used to predict the relationship between the FRPGs and the infiltration level of immune cells, and it was found that the FRPGs were closely related to the infiltration of macrophages, B cells, T cells, dendritic cells, and neutrophils (Fig. 4G). The CIBERSORT algorithm indicated that Macrophases-M1 (*p* < 0.01) and Macrophases-M2 (*p* < 0.001) were significantly higher in high risk groups than in low risk groups, while Dendritic-cells-activated was significantly higher in low risk groups than in high risk groups (Fig. 4H), suggesting the TIME and immune status of the two groups differed significantly. These results indicated that the risk signature constructed had a strong potential for prognosis prediction of GC patients, and it was significantly correlated with TIME in GC. The HPA database examined the immunohistochemical (IHC) staining of FRPGs (Fig. 5A–J), and found that the protein expressions of ZFP36, TUBE1, NFE2L2, GCH1, GABARAPL2, CHAC1, CAPG, ACSL4, ACO1, and SLC1A4 in GC and normal tissues were significantly different, there was no protein expression of NOX5, MIR9-3, and NOX4 in HPA. In addition, we observed mutations of FRPGs in the training cohort and found that TUBE1 was a dominant gene and therefore better targeted (Fig. 6).

### Validation of prognostic risk signature

**Figure 7:**
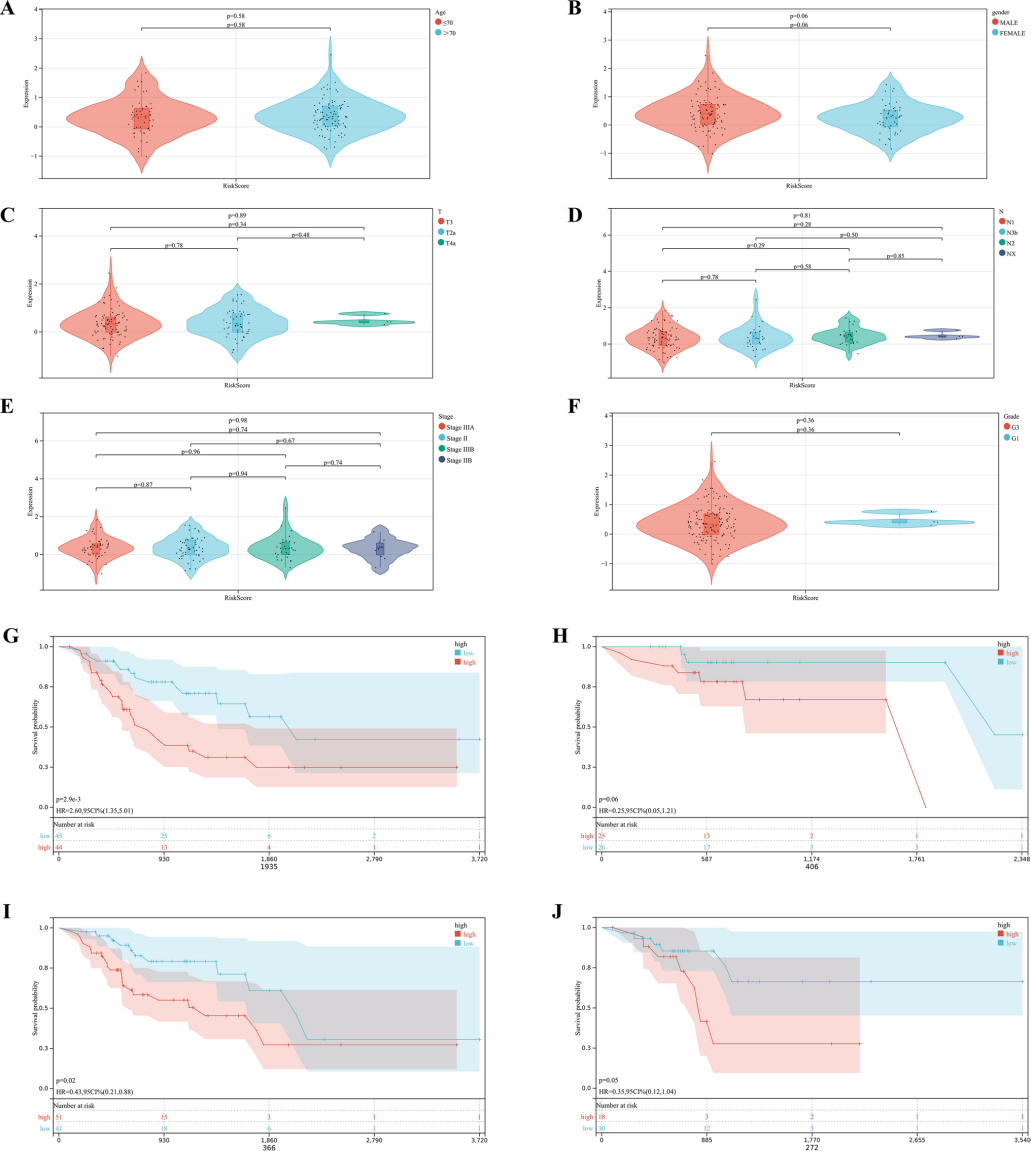
Association of risk score and clinical characteristics. Differences in risk scores among (**A**) Age, (**B**) Gender, (**C**) T, (**D**) N, (**E**) Stage, and (**F**) Grade. (**G**,**H**) Survival curve of GC patients regrouped according to age. (**I**,**J**) Survival curve of GC patients regrouped according to gender.

We assessed the differences in risk scores among the subgroups by age (Fig. 7A), gender (Fig. 7B), T (Fig. 7C), N (Fig. 7D), Stage (Fig. 7E), and Grade (Fig. 7F) and found no significant differences between the subgroups, indicating that the risk scores were not correlated with the clinical characteristics of the patient. Besides, when the patients were regrouped according to age (Fig. 7G,H), and gender (Fig. 7I,J), the risk signature still exhibited potent predictive performance and those patients with lower risk score enjoyed better prognosis. This indicated that the prognosis signature we constructed can be used to independently predict the prognosis of GC patients.

**Figure 8:**
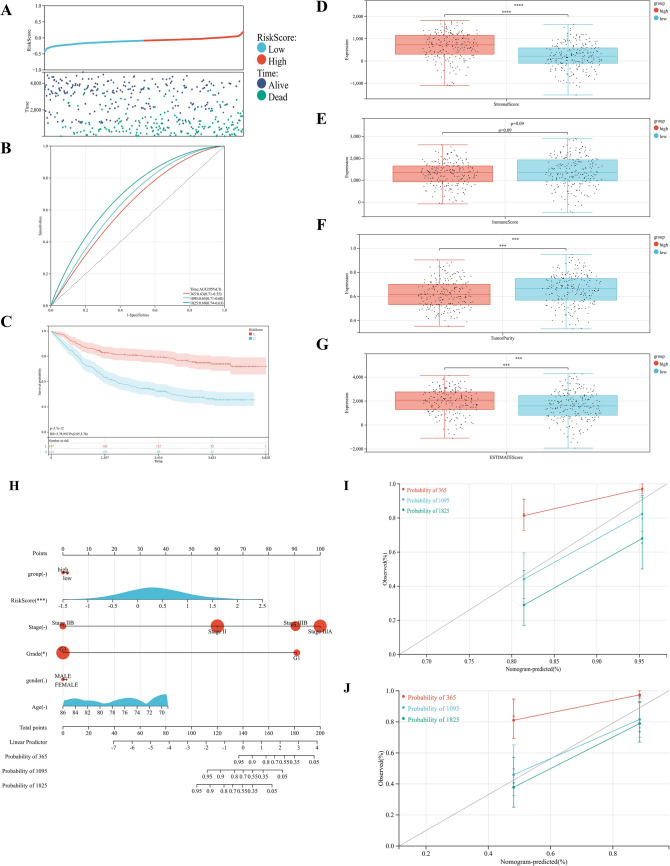
Validation of prognostic risk signature in validation cohort. (**A**) Distribution of survival status and risk score in the high and low risk groups. (**B**) ROC curve of the risk signature in validation cohort. (**C**) Survival curve of the patients in the high and low risk groups. (**D**–**G**) Stromal score, Immune score, Tumor Purity, and ESTIMATE score calculated by ESTIMATE algorithm (*p < 0.05; **p < 0.01; ***p < 0.001). (**H**) Nomogram integrating risk score and clinical features. (**I**) Calibration of the nomogram at 1,3, and 5 years in the training cohort (*p < 0.05; **p < 0.01; ***p < 0.001). (**J**) Calibration of the nomogram at 1,3, and 5 years in the validation cohort (*p < 0.05; **p < 0.01; ***p < 0.001).

To verify the stability of the prognostic signature developed in the training cohort, we calculated the risk score for each patient in the validation cohort using the same formula as in the build training cohort. Patients in the validation cohort were grouped into high (n = 216) and low risk (n = 293) groups (Fig. 8A), and ROC analysis revealed AUC values of 0.63, 0.65, and 0.69 at 1, 3, and 5 years, respectively (Fig. 8B). The Survival curve showed that the low risk group had a better prognosis than the high risk group (Fig. 8C). In addition, the ESTIMATE algorithm was performed on high risk and low risk group in the validation cohort, and the results showed that the high risk group had higher Stromal score (*p* < 0.001), higher ESTIMATE score (*p* < 0.001), and lower Tumor Purity (*p* < 0.001) (Fig. 8D–G). These results demonstrated that the established risk signature was correlated with TIME and prognosis in GC in the validation cohort.

Finally, a nomogram integrating the risk signature and clinical features were constructed to predict the prognosis of the GC patients more precisely (Fig. 8H). The 3 years survival rates observed with the nomogram were matched well to the actual survival rates (Fig. 8I), and similar result was also observed in the validation cohort (Fig. 8J), demonstrating that the nomogram could predict the prognosis of GC patients accurately.

### Functional enrichment analysis

**Figure 9:**
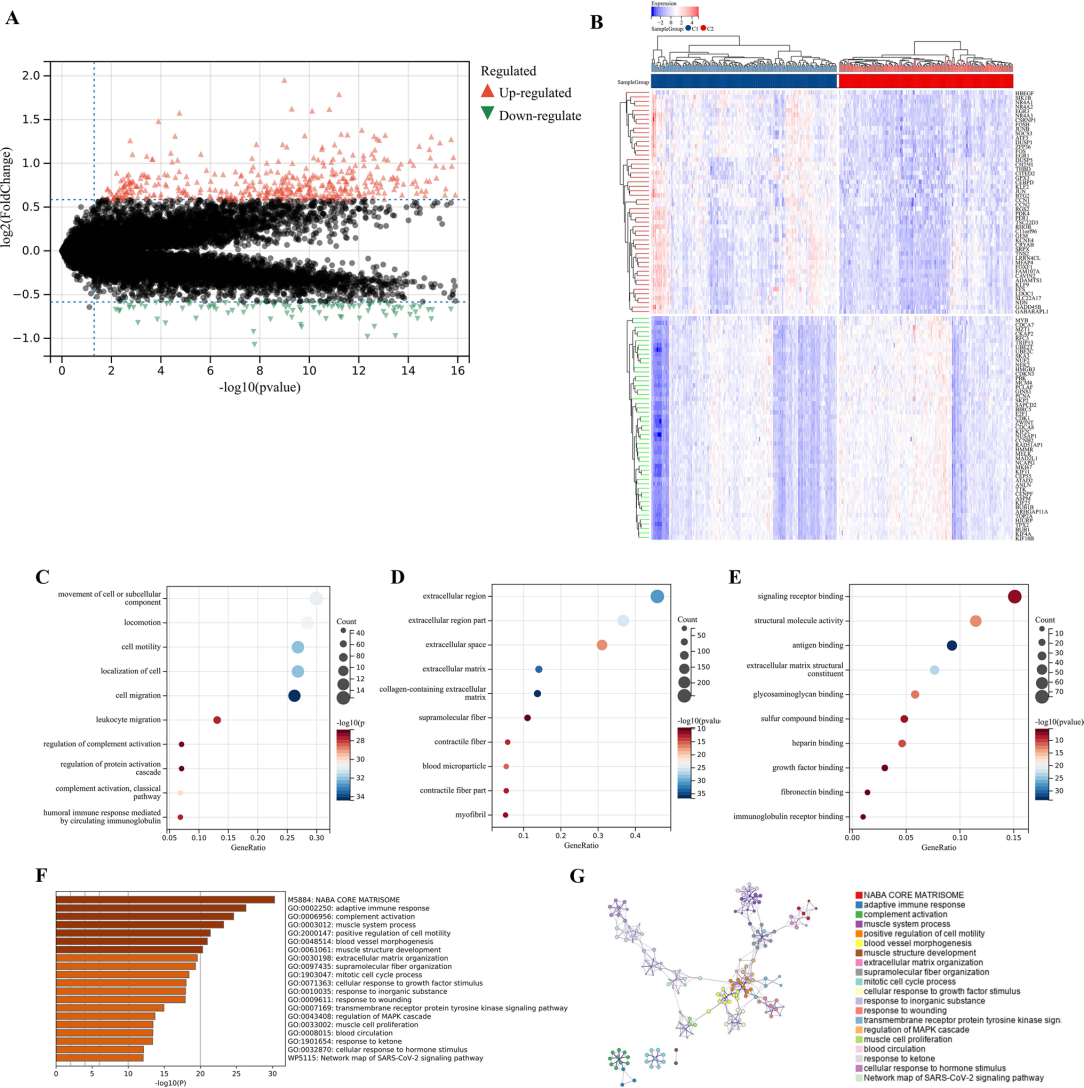

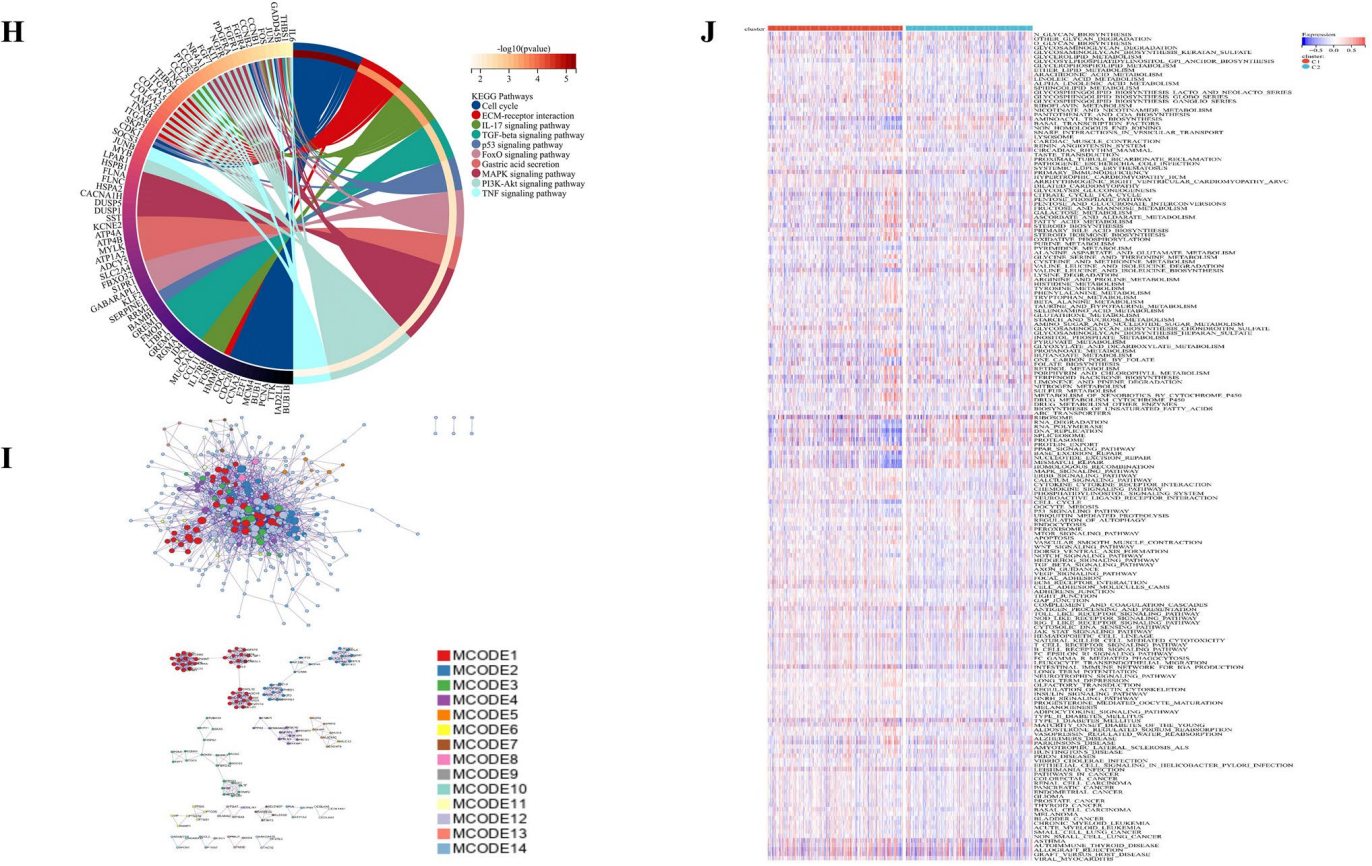
Identification of DEGs and Functional Enrichment Analysis. (**A**) Volcanic map of DEGs in training cohort. (**B**) Heatmap of DEGs in training cohort. (**C**–**F**) BP, CC, MF, and the whole gene ontology (GO) Analysis of DEGs in training cohort. (**G**) PPI analysis of GO Analysis in training cohort. (**H**) Circle diagram of KEGG in training cohort. (**I**) PPI analysis of KEGG enrichment analysis. (**J**) Heatmap of GSEA analysis results.

A total of 570 DEGs were detected compared to Cluster C2, of which 465 genes were up regulated and 105 genes were down regulated in Cluster C1 (Fig. 9A,B). GO enrichment analysis showed that the biological process (BP) of DEGs mainly included cell migration, immune globulin, human immune response mediated by circulating immune globulin. Cellular component (CC) was mainly enriched in the extracellular matrix, and extracellular region part. Molecular function (MF) mainly included immunoglobulin receptor binding, fibronectin binding, and growth factor binding (Fig. 9C–G). The KEGG enrichment analysis showed that DEGs was mainly enriched in the Cell cycle, p53 signaling pathway, IL-17 signaling pathway, MAPK signaling pathway, and PI3K-Akt signaling pathway (Fig. 9H–I). To further explore the relationship between enrichment pathways and prognosis of GC patients, we performed GSEA analysis, and the results showed that compared with Cluster C2, Cluster C1 showed lower expression in lipid metabolism and glutathione metabolism, which might be related to the poor prognosis of GC patients (Fig. 9J). All these results demonstrated that expression of FRPGs were correlated with immunity and ferroptosis, which may be involved in the poor prognosis of GC patients.

**Figure 10:**
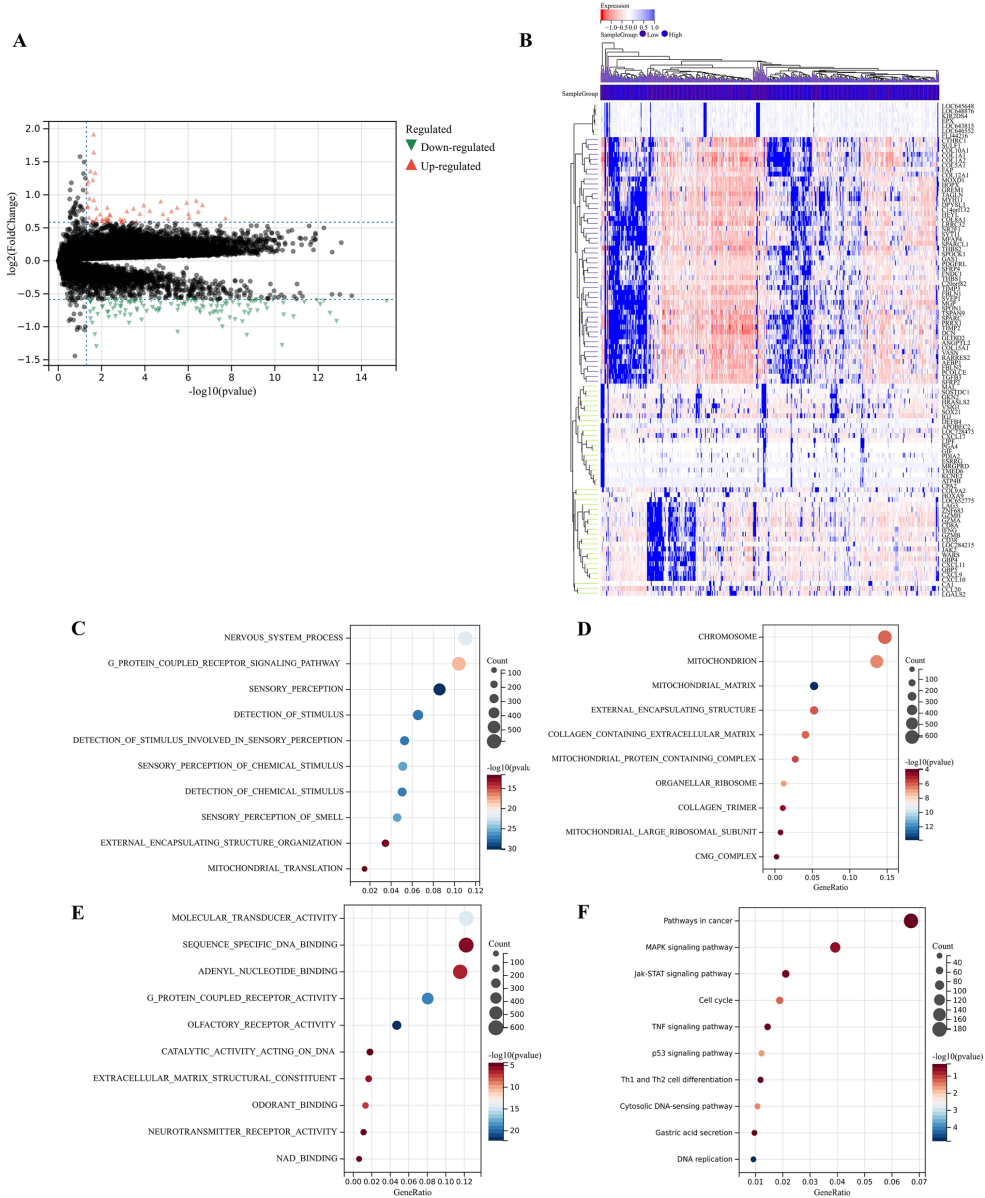
Identification of DEGs and functional enrichment analysis in GEO cohorts (www.kegg.jp/kegg/kegg1.html). (**A**) The volcanic map of DEGs. (**B**) The heatmap of DEGs. (**C**–**F**) The BP, CC, MF, and the whole GO of DEGs.

Compared with the DEGs in training cohort, a total of 71 DEGs were detected, of which 7 genes were up regulated and 64 genes were down regulated in validation cohort (Fig. 10A,B). KEGG enrichment analysis showed that DEGs was mainly enriched in the Cell cycle, p53 signaling pathway, and MAPK signaling pathway, which consistent with the results in training cohort. In addition, DEGs in validation cohort was also enriched in the Jak-STAT signaling pathway, TNF signaling pathway (Fig. 10C–F).

### Quantitative Real-Time PCR

**Figure 11:**
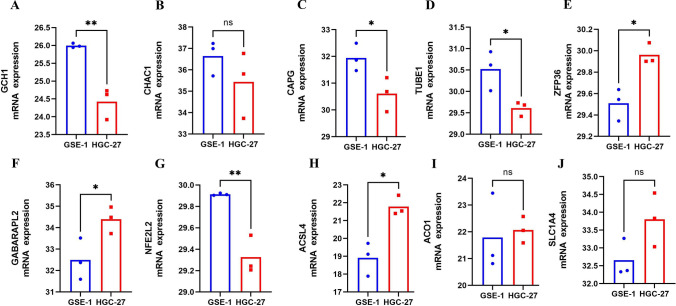
mRNA expression level of FRPGs. mRNA expression level of (**A**) GCH1, (**B**) CHAC1, (**C**) CAPG, (**D**) TUBE1, (**E**) ZFP36, (**F**) GABARAPL2, (**G**) NFE2L2, (**H**) ACSL4, (**I**) ACO1, and (**J**) SLC1A4 by qRT-PCR (*p < 0.05; **p < 0.01; ***p < 0.001).

We found that the protein expressions of ZFP36, TUBE1, NFE2L2, GCH1, GABARAPL2, CHAC1, CAPG, ACSL4, ACO1, and SLC1A4 in GC and normal tissues were significantly different. Their expression levels were evaluated in GES-1 and HGC-27 by qRT-PCR. Consistently, compared with GES-1, GCH1 (*p* < 0.01), CAPG (*p* < 0.05), and TUBE1 (*p* < 0.05) were significantly upregulated in HGC-27, while ZFP36 (*p* < 0.05), GABARAPL2 (*p* < 0.05), NFE2L2 (*p* < 0.01), and ACSL4 (*p* < 0.05) were downregulated, but CHAC1, ACO1, and SLC1A4 were no significant difference (Fig. 11A–J). In summary, the potential roles of the FRPGs could also be verified in cell line experiment.

## Discussion

In recent years, various prognostic signatures have been proposed to predict the prognosis and immune infiltration of malignant tumors^35^. Including predicting the immune status and prognosis of malignant tumors by screening immune-related genes or FRGs. Few studies have elucidated the effects of the FRGs on TIME and prognosis. Therefore, in order to further verify the effect of ferroptosis on TIME in GC and explore the prognostic value of FRPGs in patients with GC, we constructed a prognostic risk signature based on FRPGs and validated it in the validation cohort.

Firstly, we divided GC patients into two subgroups by consistent clustering. Cluster C2 showed a significant median survival advantage, while Cluster C1 showed a poor prognosis. Subsequently, we performed an immune analysis to explore the role of ferroptosis in TIME. TIME plays a vital role in the prognosis of patients because tumor progression is associated with changes in the surrounding matrix, of which immune cells are a key component^36^. Therefore, we applied ESTIMATE, MCPCounter, TIMER, and CIBERSORT algorithms to determinate the TIME of the two subgroups. Compared with Cluster C1, GC patients in Cluster C1 had significantly higher ESTIMATE scores, higher Immune score, and higher Stromal score. Our results indicated that Macrophases-M1, CD4 Memory Activated-T cells, NK cells, fibrolasts, CD4 + T cells, B cells were significantly higher in Cluster C1 than in Cluster C2. Macrophases-M2 and Tregs in two Cluster was no significant difference, suggesting a relatively high immune status in Cluster C1. M1 macrophages are linked to antitumor activity, whereas M2 macrophages are associated with cancer progression and metastasis^37^. These results demonstrated that the TIME and immune status of the two molecular subtypes differed significantly. TIME is generally divided into three categories: immune inflammation, immune rejection and immune desert^38^. In this study, according to the exhibitions that the TIME of the patients in the Cluster C1 with a higher abundance of immune cell infiltration but lower probability in response to immunotherapy, it was reasonable to speculate that Cluster C1 was in accordance with the immune rejection type. As a successful immunotherapy depends on the ability of innate and adaptive immune cells to penetrate into the tumor parenchyma and eradicate cancer cells. In addition, we also found that the high infiltration of immune cells was accompanied by the activation of the stroma, which could exclude the entry of CD8 + T cells from the tumor parenchyma to the peritumoral stroma rich in fibroblasts and collagen^39^. We speculated that the activation of the stroma might be one of the reasons for the poor prognosis of Cluster C1, which had high infiltration of immune cells. Therefore, the prognosis of high risk group was often poor.

In order to further verify the effect of ferroptosis on TIME in GC and explore the prognostic value of FRPGs in patients with GC, we constructed a prognostic risk signature based on FRPGs and validated it in the validation cohort. The genes used to establish the risk signature in this study have been shown to be closely related to tumor development and progression. A study had shown that autophagy promotes ferroptosis by degrading anti-ferroptosis factors^40^, and ZFP36 was a key protein for autophagy and considered to be related to ferroptosis^41^. NFE2L2, a known transcription factor involved in the encoding of GC development, is overexpressed as a prognostic marker of GC^42^. OS rate in GC patients with NRF2 positive expression was significantly reduced^43^. The experiment conducted by Wei^44^ proved that GCH1 induced immunosuppression through a 5-HTP-AHR-ID01-dependent mechanism, and that the combination of metabolic intervention and immunotherapy of this pathway might be a promising strategy for the treatment of triple-negative breast cancer (TNBC), and the GCH1 inhibitor could be used as an analgesic^45^. Members of the GABARAP family (GABARAP, GABARAPL1/GEC1 and GABARAPL2/GATE-16) are one of the subfamilies of the ATG8 protein family, which are related to the receptor and autophagy pathway^46^. The high-expression of GABARAP is related to the good prognosis of tumors^47^. CHAC1 is an enzyme related to the activity of γ-glutamyl cyclotransferase that can degrade intracellular GSH and promote ferroptosis of tumor cells^48^, which has been proved to be related to glioma^49^ and breast cancer^50^. CAPG is particularly abundant in macrophage expression^51^, and CAPG had been proved to be related to tumor cell invasion and tumorigenic^52^. SLC1A4 is one of the members of solute carrier family 1(SLC1), and SLC1A4 is one of the important roles of amino acid transporter^53^. SLC1A4 is highly expressed in pancreatic ductal adenocarcinoma and liver cancer cells, and some studies have suggested that SLC1A4 may promote the process of ferroptosis^54^. ACSL4, a long-chain fatty acyl coenzyme, is closely related to the proliferation and migration of tumor cells^55^. ACSL4 had been shown to be overexpressed in breast cancer^56^, GC^57^, and liver cancer^58^. ACO1(Cytoplasmic aconitic acid hydratase) is a protein that participates in cytoplasmic and mitochondrial metabolism and, when down regulated, leads to cell death^59^. NOX is a family of encoded oxidases, NOX4 is a catalytic subunit of nicotinamide adenine dinucleotide phosphate (NADPH) oxidase complex, and NOX5 mainly encodes calcium-dependent NADPH oxidase, produces superoxide, and acts as a calcium-dependent proton channel. The ROS produced by NOX4 is involved in a variety of biological functions, including signal transduction, cell differentiation and tumor cell growth^60,61^, and NOX4 plays an important role in the process of ferroptosis^62^. Inhibition of NOX4 can significantly block ferroptosis^63^. MiRNA plays an important role in tumors. MiR-9 is overexpressed in lung cancer tissues^64^, and MiR-9 acts as a biomarker for poor prognosis in lung cancer and thyroid papillary carcinoma^65^. There is no report about the relationship between TUBE1 and tumor in the literature.

Survival analysis indicated that whether in training cohort or the validation cohort, the established risk signature showed effective predictive performance for the survival of GC patients. The ROC curve showed the reliability and stability of the risk signature. And the high risk groups were accompanied with higher Stromal score, higher ESTIMATE score, and lower Tumor Purity. The HPA database examined the IHC staining of FRPGs, and found that the protein expressions of ZFP36, TUBE1, NFE2L2, GCH1, GABARAPL2, CHAC1, CAPG, ACSL4, ACO1, and SLC1A4 in GC and normal tissues were significantly different. Finally, a nomogram integrating the risk score and clinical features was also established and calibrated, and it showed considerable property for predicting the survival. All these results confirmed the prognostic prediction role of FRPGs in GC and correlation between FRPGs and TIME.

Finally, functional analysis was performed to explore potential biological mechanisms. GO enrichment analysis showed that the biological process of DEGs in training cohort mainly included immune globulin, and human immune response mediated by circulating immune globulin. However, the detailed relationship between ferroptosis and immunity is still unclear. Therefore, we performed GSEA analysis to further elucidate the underlying mechanisms, and the results showed that compared with Cluster C2, Cluster C1 showed lower expression in lipid metabolism, which might be related to the poor prognosis of GC patients. These results suggested that the down regulated lipid metabolism resulted in the impairment of TIME, thereby leading to the poor prognosis in GC. Ferroptosis is a regulated oxidative form of cell death associated with the accumulation of lipid ROS due to enhanced lipid peroxidation^66^. Since Cluster C1 was associated with relatively low levels of ROS, a small proportion of cells die from ferroptosis. Therefore, Cluster C1 had a poor prognosis. Researchers identified that ferroptosis was related to the immune response process^67^. It is unclear whether and how ferroptosis is involved in T cell immunity and cancer immunotherapy. Studies have shown that immunotherapy-activated CD8 + T cells enhance the specific lipid peroxidation of ferroptosis in tumor cells and that increased ferroptosis contributes to the anti-tumor efficacy of immunotherapy^68^. CD8 + T cells and fatty acids orchestrate tumor ferroptosis and immunity via ACSL4. Clinically, tumor ACSL4 correlates with T cell signatures and improved survival in ICB-treated cancer patients^69^.

KEGG enrichment analysis showed that DEGs in training cohort were mainly enriched in the p53 signaling pathway, IL-17 signaling pathway, MAPK signaling pathway, and PI3K-Akt signaling pathway. Studies have found that these pathways are associated with immune response^70–73^. p53 is a tumor suppressor gene, and p53 mutations have been reported in many cancers^74^. When p53 mutations occur, cells proliferate abnormally and transform into cancer cells. GC patients with p53 mutation have worse prognosis than those without mutation^75^. More and more evidences support the pathogenic role of IL-17 in cancer formation, including colon cancer^76^ and lung cancer^77^. Wu^78^ found that IL-17 could promote tumor angiogenesis by mediating the up-regulation of VEGF in GC through STAT3 pathway. It has been confirmed that MAPK and PI3K-Akt pathways are involved in many processes of the occurrence and development of GC^79–81^.

Although multiple studies have established relevant prognostic models for ferroptosis in GC^82,83^, our study shows unique advantages compared with previous studies. Firstly, the number of patients was significantly different from that of published article. Secondly, our work focused on ferroptosis in patients with GC and identified two significantly different molecular subtypes of prognosis and immune status by consensus clustering. Thirdly, genes were obtained in different ways, we have selected differential genes based on molecular subtypes and partially elucidated the underlying mechanisms. Fourthly, we used the GEO data set to validate the prognosis model. Fifthly, we elucidated the effects of ferroptosis on TIME and prognosis. Sixthly, we verified the mRNA expression of FRPGs by qRT-PCR. Seventhly, the prognostic model based on FRPGs we constructed different from the previous articles. Finally, we speculated that the down regulated lipid metabolism may result in the impairment of TIME, thereby leading to the poor prognosis in GC. The establishment of prognostic risk signature based on FRPGs provided new possibilities for us to predict the efficacy of immunotherapy, and promotes personalized treatment for GC patients in the future. However, this study has certain limitations. Our signature was constructed and validated based on retrospective data, without relevant clinical experimental verification.

## Conclusion

In summary, in this study, we identified two molecular subtypes, Clusters C1 and C2. In Cluster C1, patients with poor prognosis present with a hyperimmune state and low lipid metabolism, and we speculated that Cluster C1 was in accordance with the immune rejection type. The risk model based on FRPGs can accurately predict the prognosis of GC. These results indicated that ferroptosis is associated with TIME, and the down regulated lipid metabolism may result in the impairment of TIME, thereby leading to the poor prognosis in GC.

## Supplementary Information

Supplementary Information 1.

Supplementary Information 2.

## Data Availability

The data used to support the findings of this study are included within the article.

## Abbreviations

TCGA: The Cancer Genome Atlas
GEO: Gene Expression Omnibus
GC: Gastric cancer
FRGs: Ferroptosis-related genes
GSEA: Gene set enrichment analysis
FRPGs: Ferroptosis-related prognosis genes
TIME: Tumor immune microenvironment
TIIC: Tumor infiltrating immune cells
HPA: Human protein atlas
IHC: Immunohistochemical
DEGs: Differentially expressed genes
GO: Gene ontology
BP: Biological process
CC: Cellular component
MF: Molecular function
FDR: Error detection rate
KEGG: Kyoto Encyclopedia of Genes and Genomes
PPI: Protein–protein interaction
OS: Overall survival
