# Supplementary material for: Expression of ferroptosis-related gene correlates with immune microenvironment and predicts prognosis in gastric cancer
Source: Sci Rep. 2022 May 24;12:8785. doi: 10.1038/s41598-022-12800-6 (PMC9129902; doi:10.1038/s41598-022-12800-6)
Supplement: Supplementary file 1 — Supplementary Information 1. [file 41598_2022_12800_MOESM1_ESM.docx]

**Supplementary Table S1 Real-Time polymerase chain reaction primers**

| **Gene** | **Sequence** **(5′-3′)** |
| --- | --- |
| ZFP36-F | TGTCTCCTGGTAACTGGAACCTCTC |
| ZFP36-R | TGGCAACGGCTTTGGCTACTTG |
| TUBE1-F | GCCAGTCCCTCAGGTTCATAATGC |
| TUBE1-R | TAGGTGGTCAGATGGGCTCACTTAC |
| NFE2L2-F | TGTGGCAGGTGAATTGGAAGATGG |
| NFE2L2-R  GCH1-F  GCH1-R  GABARAPL2-F  GABARAPL2-R  CHAC1-F  CHAC1-R  CAPG -F  CAPG-R  ACSL4-F  ACSL4-R  ACO1-F  ACO1-R  SLC1A4-F  SLC1A4-R  β-ACTIN-F | CCAACTAAGCCGTCACAACAATGC  ATAATGGGATGGCTGGGTCAAATGG  GGTGCTGGAGAGAATGTGGAGAAAC  TCACCTCGCTGTCACCTCTGTC  CTGTAACTGGGACTGGAACCTCAAC  TGATTAGCATTGGAGGCAGGACTTG  ACAGGCATTACCCGCAGAGAGG  AGGAGATTGGCTGGCTGAGGAG  CTGGTAATGGCGAGGTCTGTTCAC  TCTCTTGCCTCAGCCTCCTTAGTAG  CGAGACCAGCCTGACCAACATG  TCCAACAGCAGCATCGTCAGAAC  GCATACATCCAGTAAGAGGCAGAGC  TGTGTGCTTTGGTCTGCTCATCTG  CCGTGCTAACTCGCTCAATCTTCC  CAGATGTGGATCAGCAAGCAGGA |
| β-ACTIN-R | CGCAACTAAGTCATAGTCCGCCTA |
